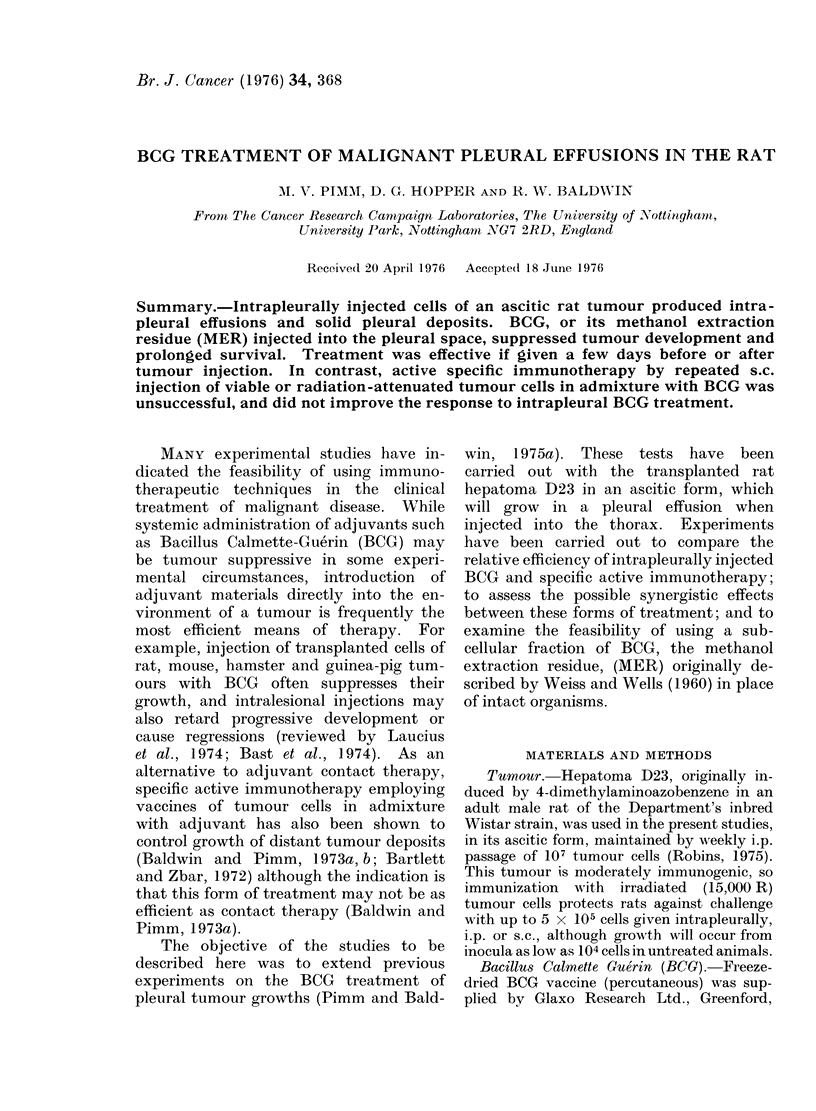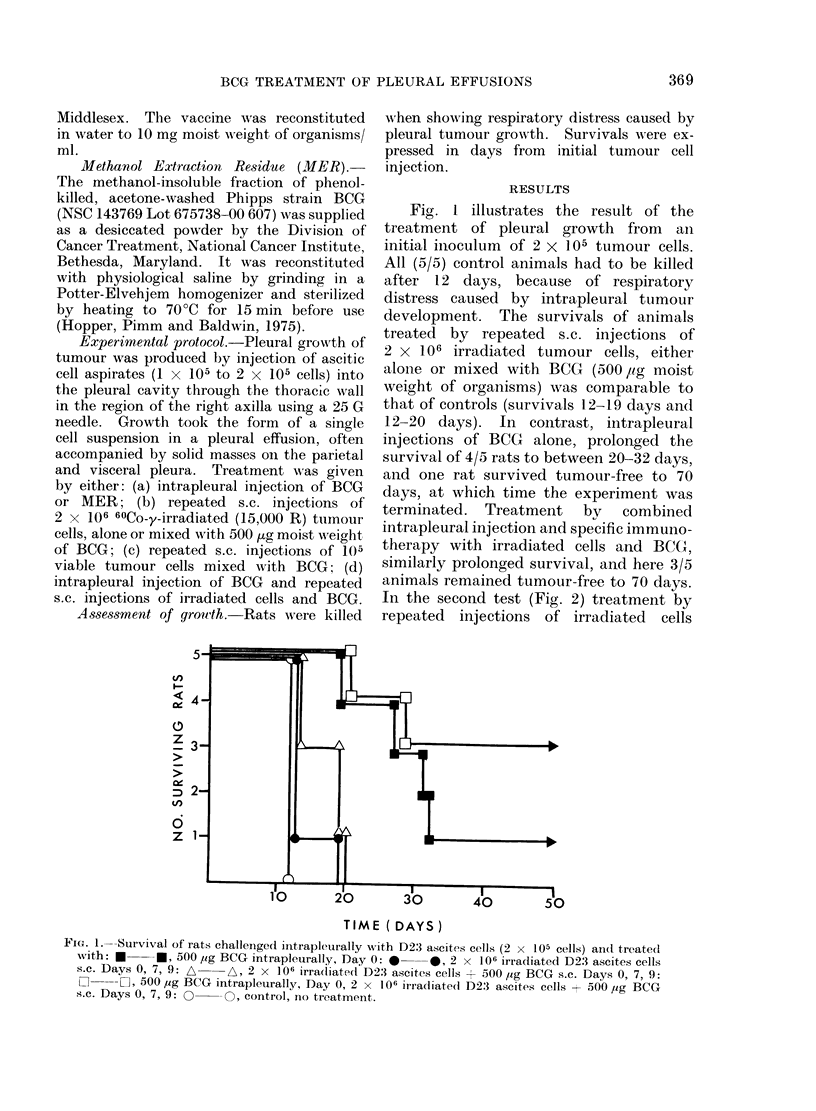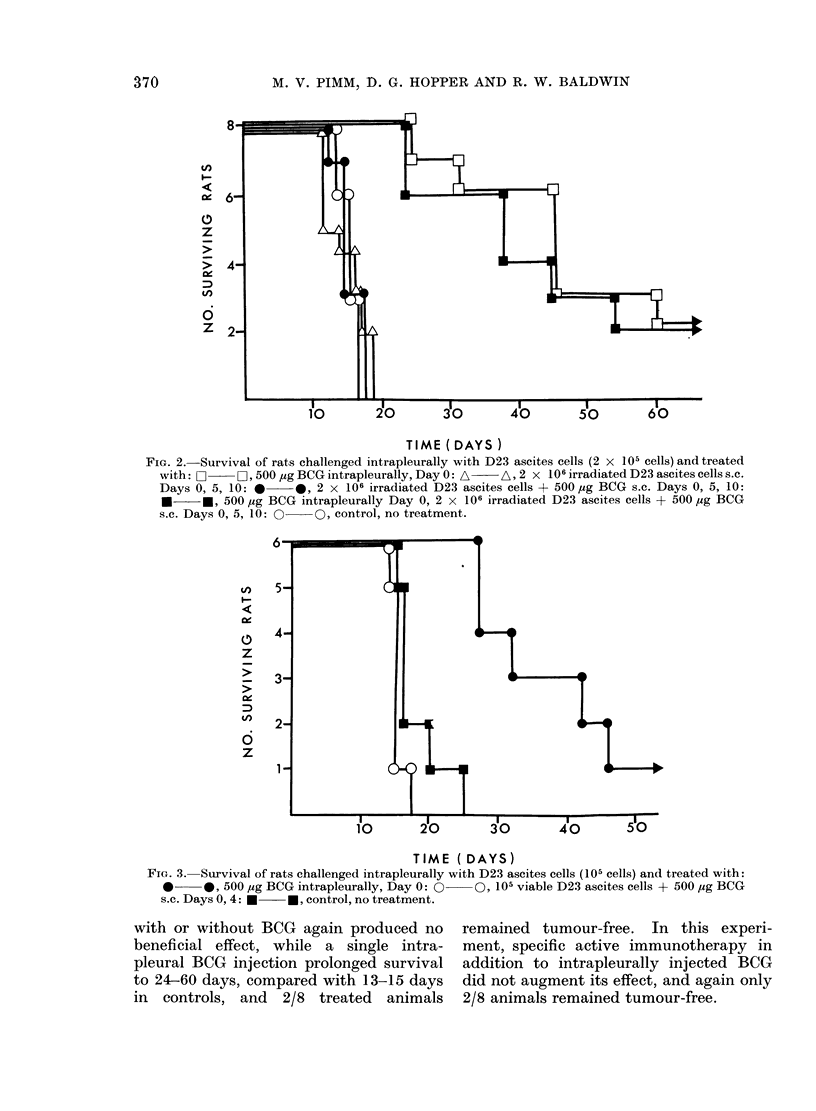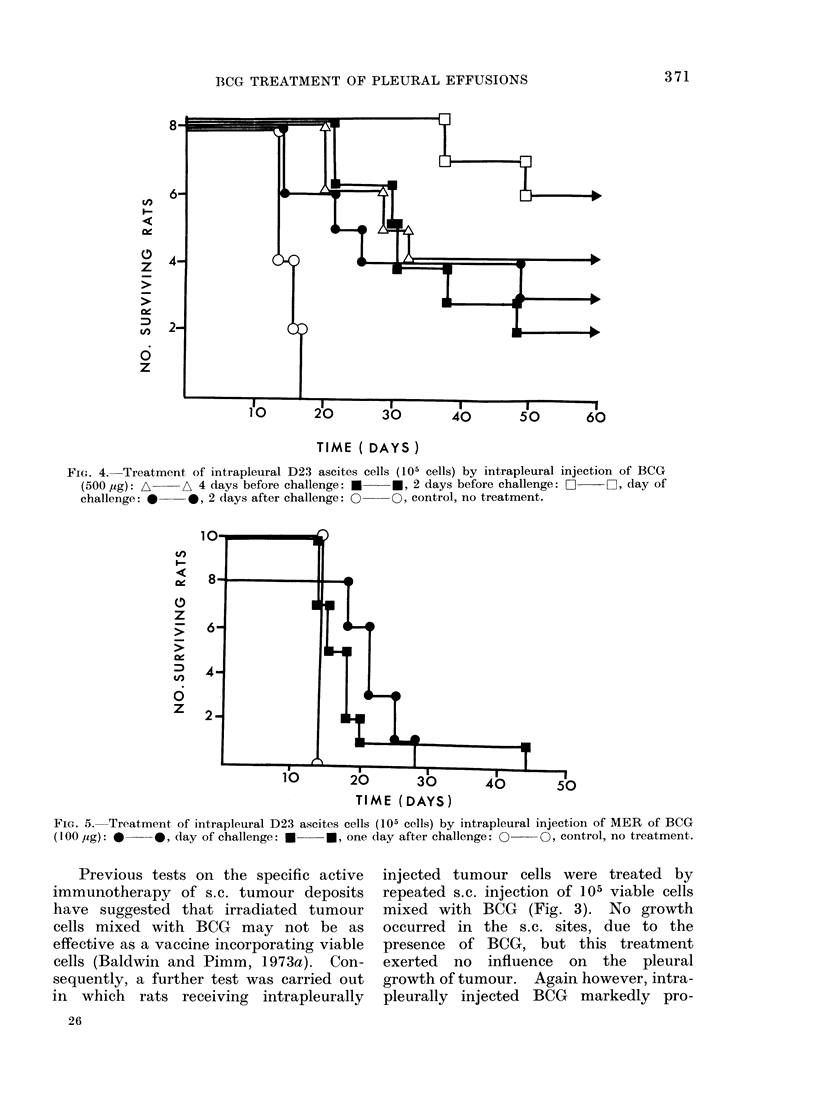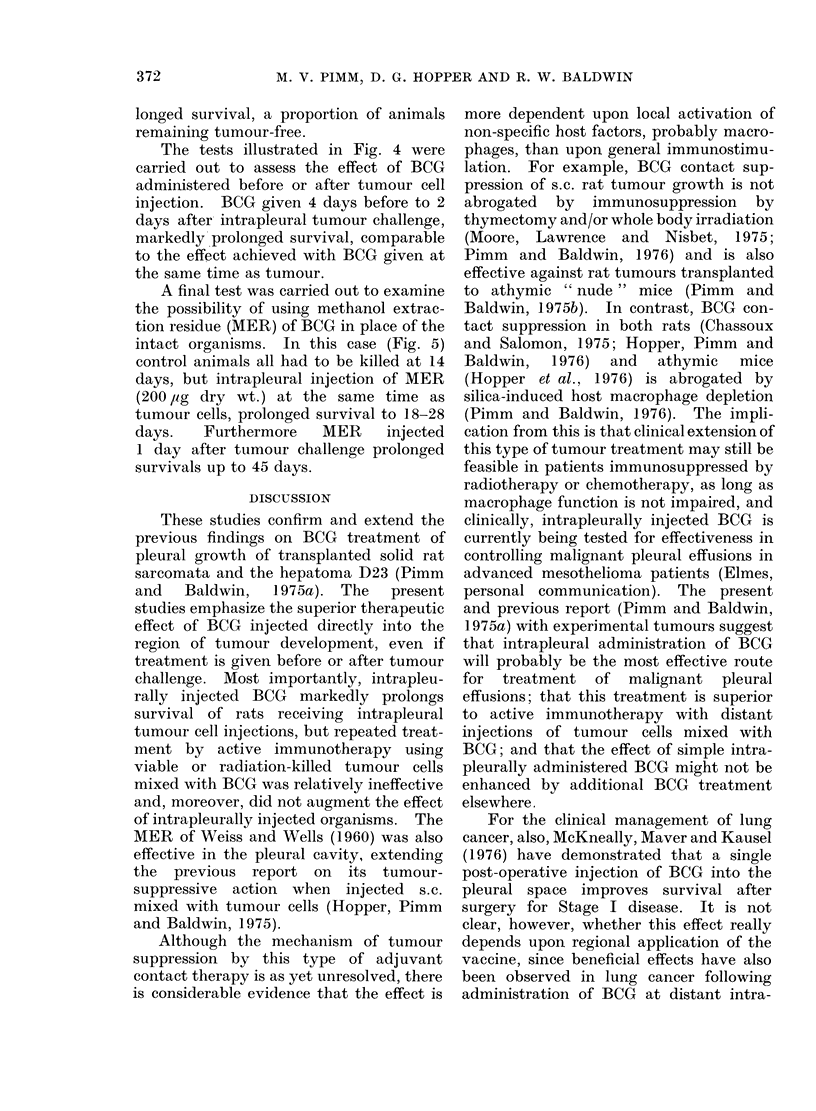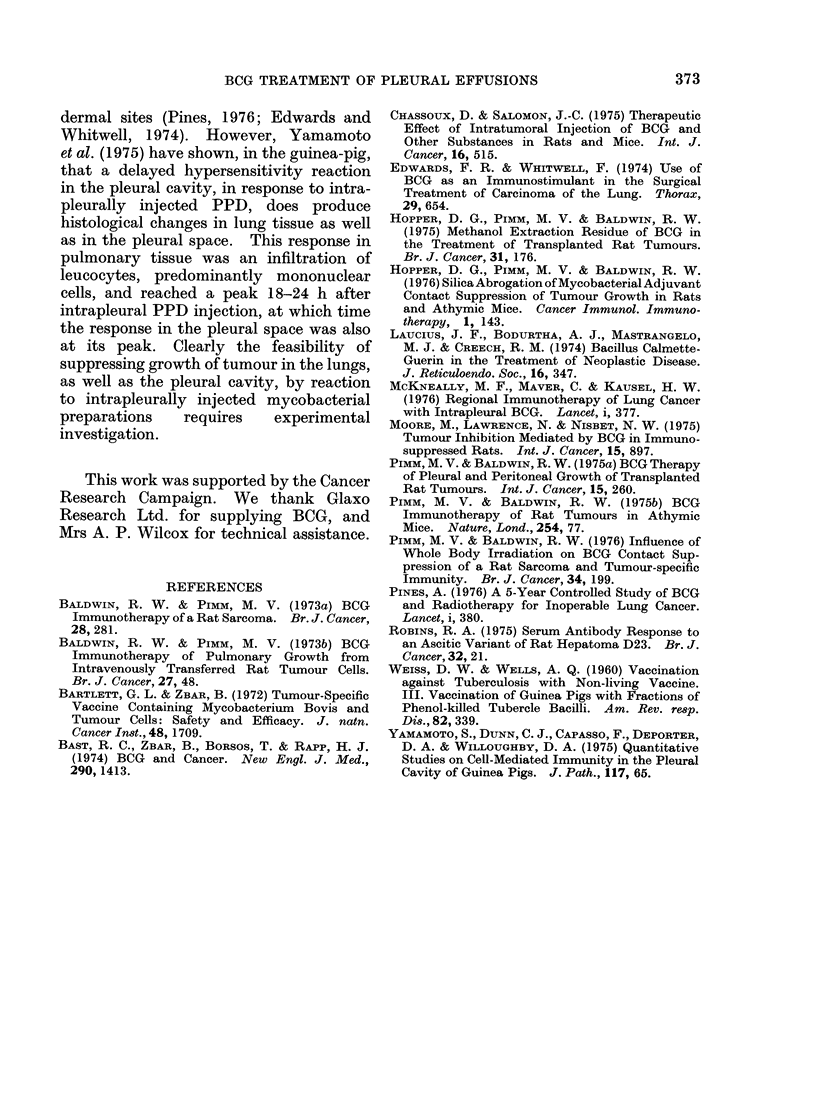# BCG treatment of malignant pleural effusions in the rat.

**DOI:** 10.1038/bjc.1976.179

**Published:** 1976-10

**Authors:** M. V. Pimm, D. G. Hopper, R. W. Baldwin

## Abstract

Intrapleurally injected cells of an ascitic rat tumour produced intrapleural effusions and solid pleural deposits. BCG, or its methanol extraction residue (MER) injected into the pleural space, suppressed tumour development and prolonged survival. Treatment was effective if given a few days before or after tumour injection. In contrast, active specific immunotherapy by repeated s.c. injection of viable or radiation-attenuated tumour cells in admixture with BCG was unsuccessful, and did not improve the response to intrapleural BCG treatment.


*Br. J. Cancer* (1976) **34**, 368

### BCG TREATMENT OF MALIGNANT PLEURAL EFFUSIONS IN THE RAT

M. V. PIMM, D. G. HOPPER AND R. W. BALDWIN

*From The Cancer Research Campaign Laboratories, The University of Nottingham, University Park, Nottingham NG7 2RD, England*

Received 20 April 1976 Accepted 18 June 1976

**Summary.—Intrapleurally injected cells of an ascitic rat tumour produced intrapleural effusions and solid pleural deposits. BCG, or its methanol extraction residue (MER) injected into the pleural space, suppressed tumour development and prolonged survival. Treatment was effective if given a few days before or after tumour injection. In contrast, active specific immunotherapy by repeated s.c. injection of viable or radiation-attenuated tumour cells in admixture with BCG was unsuccessful, and did not improve the response to intrapleural BCG treatment.**

MANY experimental studies have indicated the feasibility of using immunotherapeutic techniques in the clinical treatment of malignant disease. While systemic administration of adjuvants such as Bacillus Calmette-Guérin (BCG) may be tumour suppressive in some experimental circumstances, introduction of adjuvant materials directly into the environment of a tumour is frequently the most efficient means of therapy. For example, injection of transplanted cells of rat, mouse, hamster and guinea-pig tumours with BCG often suppresses their growth, and intralesional injections may also retard progressive development or cause regressions (reviewed by Laucius *et al.*, 1974; Bast *et al.*, 1974). As an alternative to adjuvant contact therapy, specific active immunotherapy employing vaccines of tumour cells in admixture with adjuvant has also been shown to control growth of distant tumour deposits (Baldwin and Pimm, 1973*a*, *b*; Bartlett and Zbar, 1972) although the indication is that this form of treatment may not be as efficient as contact therapy (Baldwin and Pimm, 1973*a*).

The objective of the studies to be described here was to extend previous experiments on the BCG treatment of pleural tumour growths (Pimm and Baldwin, 1975*a*). These tests have been carried out with the transplanted rat hepatoma D23 in an ascitic form, which will grow in a pleural effusion when injected into the thorax. Experiments have been carried out to compare the relative efficiency of intrapleurally injected BCG and specific active immunotherapy; to assess the possible synergistic effects between these forms of treatment; and to examine the feasibility of using a subcellular fraction of BCG, the methanol extraction residue, (MER) originally described by Weiss and Wells (1960) in place of intact organisms.

### MATERIALS AND METHODS

*Tumour.*—Hepatoma D23, originally induced by 4-dimethylaminoazobenzene in an adult male rat of the Department's inbred Wistar strain, was used in the present studies, in its ascitic form, maintained by weekly i.p. passage of $10^7$ tumour cells (Robins, 1975). This tumour is moderately immunogenic, so immunization with irradiated (15,000 R) tumour cells protects rats against challenge with up to $5 \times 10^5$ cells given intrapleurally, i.p. or s.c., although growth will occur from inocula as low as $10^4$ cells in untreated animals.

*Bacillus Calmette Guérin* (*BCG*).—Freeze-dried BCG vaccine (percutaneous) was supplied by Glaxo Research Ltd., Greenford,

Middlesex. The vaccine was reconstituted in water to 10 mg moist weight of organisms/ml.

*Methanol Extraction Residue* (*MER*).—The methanol-insoluble fraction of phenol-killed, acetone-washed Phipps strain BCG (NSC 143769 Lot 675738–00 607) was supplied as a desiccated powder by the Division of Cancer Treatment, National Cancer Institute, Bethesda, Maryland. It was reconstituted with physiological saline by grinding in a Potter-Elvehjem homogenizer and sterilized by heating to 70°C for 15 min before use (Hopper, Pimm and Baldwin, 1975).

*Experimental protocol.*—Pleural growth of tumour was produced by injection of ascitic cell aspirates ($1 \times 10^5$ to $2 \times 10^5$ cells) into the pleural cavity through the thoracic wall in the region of the right axilla using a 25 G needle. Growth took the form of a single cell suspension in a pleural effusion, often accompanied by solid masses on the parietal and visceral pleura. Treatment was given by either: (a) intrapleural injection of BCG or MER; (b) repeated s.c. injections of $2 \times 10^6$ $^{60}$Co-$\gamma$-irradiated (15,000 R) tumour cells, alone or mixed with 500 $\mu$g moist weight of BCG; (c) repeated s.c. injections of $10^5$ viable tumour cells mixed with BCG; (d) intrapleural injection of BCG and repeated s.c. injections of irradiated cells and BCG.

*Assessment of growth.*—Rats were killed when showing respiratory distress caused by pleural tumour growth. Survivals were expressed in days from initial tumour cell injection.

### RESULTS

Fig. 1 illustrates the result of the treatment of pleural growth from an initial inoculum of $2 \times 10^5$ tumour cells. All (5/5) control animals had to be killed after 12 days, because of respiratory distress caused by intrapleural tumour development. The survivals of animals treated by repeated s.c. injections of $2 \times 10^6$ irradiated tumour cells, either alone or mixed with BCG (500 $\mu$g moist weight of organisms) was comparable to that of controls (survivals 12–19 days and 12–20 days). In contrast, intrapleural injections of BCG alone, prolonged the survival of 4/5 rats to between 20–32 days, and one rat survived tumour-free to 70 days, at which time the experiment was terminated. Treatment by combined intrapleural injection and specific immunotherapy with irradiated cells and BCG, similarly prolonged survival, and here 3/5 animals remained tumour-free to 70 days. In the second test (Fig. 2) treatment by repeated injections of irradiated cells

FIG. 1.—Survival of rats challenged intrapleurally with D23 ascites cells ($2 \times 10^5$ cells) and treated with: ■——■, 500 $\mu$g BCG intrapleurally, Day 0: ●——●, $2 \times 10^6$ irradiated D23 ascites cells s.c. Days 0, 7, 9: △——△, $2 \times 10^6$ irradiated D23 ascites cells + 500 $\mu$g BCG s.c. Days 0, 7, 9: □——□, 500 $\mu$g BCG intrapleurally, Day 0, $2 \times 10^6$ irradiated D23 ascites cells + 500 $\mu$g BCG s.c. Days 0, 7, 9: ○——○, control, no treatment.

FIG. 2.—Survival of rats challenged intrapleurally with D23 ascites cells (2 × $10^5$ cells) and treated with: □——□, 500 μg BCG intrapleurally, Day 0: △——△, 2 × $10^6$ irradiated D23 ascites cells s.c. Days 0, 5, 10: ●——●, 2 × $10^6$ irradiated D23 ascites cells + 500 μg BCG s.c. Days 0, 5, 10: ■——■, 500 μg BCG intrapleurally Day 0, 2 × $10^6$ irradiated D23 ascites cells + 500 μg BCG s.c. Days 0, 5, 10: ○——○, control, no treatment.

FIG. 3.—Survival of rats challenged intrapleurally with D23 ascites cells ($10^5$ cells) and treated with: ●——●, 500 μg BCG intrapleurally, Day 0: ○——○, $10^5$ viable D23 ascites cells + 500 μg BCG s.c. Days 0, 4: ■——■, control, no treatment.

with or without BCG again produced no beneficial effect, while a single intrapleural BCG injection prolonged survival to 24–60 days, compared with 13–15 days in controls, and 2/8 treated animals remained tumour-free. In this experiment, specific active immunotherapy in addition to intrapleurally injected BCG did not augment its effect, and again only 2/8 animals remained tumour-free.

FIG. 4.—Treatment of intrapleural D23 ascites cells ($10^5$ cells) by intrapleural injection of BCG (500 $\mu$g): △——△ 4 days before challenge: ■——■, 2 days before challenge: □——□, day of challenge: ●——●, 2 days after challenge: ○——○, control, no treatment.

FIG. 5.—Treatment of intrapleural D23 ascites cells ($10^5$ cells) by intrapleural injection of MER of BCG (100 $\mu$g): ●——●, day of challenge: ■——■, one day after challenge: ○——○, control, no treatment.

Previous tests on the specific active immunotherapy of s.c. tumour deposits have suggested that irradiated tumour cells mixed with BCG may not be as effective as a vaccine incorporating viable cells (Baldwin and Pimm, 1973*a*). Consequently, a further test was carried out in which rats receiving intrapleurally injected tumour cells were treated by repeated s.c. injection of $10^5$ viable cells mixed with BCG (Fig. 3). No growth occurred in the s.c. sites, due to the presence of BCG, but this treatment exerted no influence on the pleural growth of tumour. Again however, intrapleurally injected BCG markedly prolonged survival, a proportion of animals remaining tumour-free.

The tests illustrated in Fig. 4 were carried out to assess the effect of BCG administered before or after tumour cell injection. BCG given 4 days before to 2 days after intrapleural tumour challenge, markedly prolonged survival, comparable to the effect achieved with BCG given at the same time as tumour.

A final test was carried out to examine the possibility of using methanol extraction residue (MER) of BCG in place of the intact organisms. In this case (Fig. 5) control animals all had to be killed at 14 days, but intrapleural injection of MER (200 $\mu$g dry wt.) at the same time as tumour cells, prolonged survival to 18–28 days. Furthermore MER injected 1 day after tumour challenge prolonged survivals up to 45 days.

### DISCUSSION

These studies confirm and extend the previous findings on BCG treatment of pleural growth of transplanted solid rat sarcomata and the hepatoma D23 (Pimm and Baldwin, 1975*a*). The present studies emphasize the superior therapeutic effect of BCG injected directly into the region of tumour development, even if treatment is given before or after tumour challenge. Most importantly, intrapleurally injected BCG markedly prolongs survival of rats receiving intrapleural tumour cell injections, but repeated treatment by active immunotherapy using viable or radiation-killed tumour cells mixed with BCG was relatively ineffective and, moreover, did not augment the effect of intrapleurally injected organisms. The MER of Weiss and Wells (1960) was also effective in the pleural cavity, extending the previous report on its tumour-suppressive action when injected s.c. mixed with tumour cells (Hopper, Pimm and Baldwin, 1975).

Although the mechanism of tumour suppression by this type of adjuvant contact therapy is as yet unresolved, there is considerable evidence that the effect is more dependent upon local activation of non-specific host factors, probably macrophages, than upon general immunostimulation. For example, BCG contact suppression of s.c. rat tumour growth is not abrogated by immunosuppression by thymectomy and/or whole body irradiation (Moore, Lawrence and Nisbet, 1975; Pimm and Baldwin, 1976) and is also effective against rat tumours transplanted to athymic "nude" mice (Pimm and Baldwin, 1975*b*). In contrast, BCG contact suppression in both rats (Chassoux and Salomon, 1975; Hopper, Pimm and Baldwin, 1976) and athymic mice (Hopper *et al.*, 1976) is abrogated by silica-induced host macrophage depletion (Pimm and Baldwin, 1976). The implication from this is that clinical extension of this type of tumour treatment may still be feasible in patients immunosuppressed by radiotherapy or chemotherapy, as long as macrophage function is not impaired, and clinically, intrapleurally injected BCG is currently being tested for effectiveness in controlling malignant pleural effusions in advanced mesothelioma patients (Elmes, personal communication). The present and previous report (Pimm and Baldwin, 1975*a*) with experimental tumours suggest that intrapleural administration of BCG will probably be the most effective route for treatment of malignant pleural effusions; that this treatment is superior to active immunotherapy with distant injections of tumour cells mixed with BCG; and that the effect of simple intrapleurally administered BCG might not be enhanced by additional BCG treatment elsewhere.

For the clinical management of lung cancer, also, McKneally, Maver and Kausel (1976) have demonstrated that a single post-operative injection of BCG into the pleural space improves survival after surgery for Stage I disease. It is not clear, however, whether this effect really depends upon regional application of the vaccine, since beneficial effects have also been observed in lung cancer following administration of BCG at distant intradermal sites (Pines, 1976; Edwards and Whitwell, 1974). However, Yamamoto *et al.* (1975) have shown, in the guinea-pig, that a delayed hypersensitivity reaction in the pleural cavity, in response to intrapleurally injected PPD, does produce histological changes in lung tissue as well as in the pleural space. This response in pulmonary tissue was an infiltration of leucocytes, predominantly mononuclear cells, and reached a peak 18–24 h after intrapleural PPD injection, at which time the response in the pleural space was also at its peak. Clearly the feasibility of suppressing growth of tumour in the lungs, as well as the pleural cavity, by reaction to intrapleurally injected mycobacterial preparations requires experimental investigation.

This work was supported by the Cancer Research Campaign. We thank Glaxo Research Ltd. for supplying BCG, and Mrs A. P. Wilcox for technical assistance.

### REFERENCES

Baldwin, R. W. & Pimm, M. V. (1973*a*) BCG Immunotherapy of a Rat Sarcoma. *Br. J. Cancer*, **28**, 281.

Baldwin, R. W. & Pimm, M. V. (1973*b*) BCG Immunotherapy of Pulmonary Growth from Intravenously Transferred Rat Tumour Cells. *Br. J. Cancer*, **27**, 48.

Bartlett, G. L. & Zbar, B. (1972) Tumour-Specific Vaccine Containing Mycobacterium Bovis and Tumour Cells: Safety and Efficacy. *J. natn. Cancer Inst.*, **48**, 1709.

Bast, R. C., Zbar, B., Borsos, T. & Rapp, H. J. (1974) BCG and Cancer. *New Engl. J. Med.*, **290**, 1413.

Chassoux, D. & Salomon, J.-C. (1975) Therapeutic Effect of Intratumoral Injection of BCG and Other Substances in Rats and Mice. *Int. J. Cancer*, **16**, 515.

Edwards, F. R. & Whitwell, F. (1974) Use of BCG as an Immunostimulant in the Surgical Treatment of Carcinoma of the Lung. *Thorax*, **29**, 654.

Hopper, D. G., Pimm, M. V. & Baldwin, R. W. (1975) Methanol Extraction Residue of BCG in the Treatment of Transplanted Rat Tumours. *Br. J. Cancer*, **31**, 176.

Hopper, D. G., Pimm, M. V. & Baldwin, R. W. (1976) Silica Abrogation of Mycobacterial Adjuvant Contact Suppression of Tumour Growth in Rats and Athymic Mice. *Cancer Immunol. Immunotherapy*, **1**, 143.

Laucius, J. F., Bodurtha, A. J., Mastrangelo, M. J. & Creech, R. M. (1974) Bacillus Calmette-Guerin in the Treatment of Neoplastic Disease. *J. Reticuloendo. Soc.*, **16**, 347.

McKneally, M. F., Maver, C. & Kausel, H. W. (1976) Regional Immunotherapy of Lung Cancer with Intrapleural BCG. *Lancet*, i, 377.

Moore, M., Lawrence, N. & Nisbet, N. W. (1975) Tumour Inhibition Mediated by BCG in Immunosuppressed Rats. *Int. J. Cancer*, **15**, 897.

Pimm, M. V. & Baldwin, R. W. (1975*a*) BCG Therapy of Pleural and Peritoneal Growth of Transplanted Rat Tumours. *Int. J. Cancer*, **15**, 260.

Pimm, M. V. & Baldwin, R. W. (1975*b*) BCG Immunotherapy of Rat Tumours in Athymic Mice. *Nature, Lond.*, **254**, 77.

Pimm, M. V. & Baldwin, R. W. (1976) Influence of Whole Body Irradiation on BCG Contact Suppression of a Rat Sarcoma and Tumour-specific Immunity. *Br. J. Cancer*, **34**, 199.

Pines, A. (1976) A 5-Year Controlled Study of BCG and Radiotherapy for Inoperable Lung Cancer. *Lancet*, i, 380.

Robins, R. A. (1975) Serum Antibody Response to an Ascitic Variant of Rat Hepatoma D23. *Br. J. Cancer*, **32**, 21.

Weiss, D. W. & Wells, A. Q. (1960) Vaccination against Tuberculosis with Non-living Vaccine. III. Vaccination of Guinea Pigs with Fractions of Phenol-killed Tubercle Bacilli. *Am. Rev. resp. Dis.*, **82**, 339.

Yamamoto, S., Dunn, C. J., Capasso, F., Deporter, D. A. & Willoughby, D. A. (1975) Quantitative Studies on Cell-Mediated Immunity in the Pleural Cavity of Guinea Pigs. *J. Path.*, **117**, 65.